# The Shear Bond Strength of Gum-Imitating Composites with Traditional Composites—Preliminary Studies

**DOI:** 10.3390/ma19091748

**Published:** 2026-04-24

**Authors:** Lukasz Sokalski, Michal Krasowski, Małgorzata Iwona Szynkowska-Jóźwik, Aleksandra Zimon, Karolina Kopacz, Kinga Bociong

**Affiliations:** 1 Department of General Dentistry, Medical University of Lodz, 92-213 Lodz, Poland; lukasz.sokalski@student.umed.lodz.pl; 2 University Laboratory of Materials Research, Medical University of Lodz, 92-213 Lodz, Poland; michal.krasowski@umed.lodz.pl; 3 Faculty of Chemistry, Institute of General and Ecological Chemistry, Lodz University of Technology, 90-543 Lodz, Poland; malgorzata.szynkowska@p.lodz.pl (M.I.S.-J.); aleksandra.zimon@p.lodz.pl (A.Z.); 4 Faculty of Medical Science, Warsaw Medical Academy, 01-043 Warsaw, Poland; karolina.kopacz@umed.lodz.pl; 5 Department of Physiology, Pathophysiology and Clinical Immunology, Department of Clinical Physiology, Medical University of Lodz, 90-752 Lodz, Poland

**Keywords:** gum, composite, gum-imitating, gum-colored, adhesion, etching, sandblasting, SEM, bond, SBS

## Abstract

The use of resin-based composite imitating gum tissue enhances the aesthetics of fillings located below the physiological gum line. The shear bond strength (SBS) between the gum-imitating composite and the traditional composite with different surface preparation methods was examined. The aim of the study was to evaluate which base material—G-aenial Universal Injectable (GC, Japan, flow) or G-aenial A’CHORD (GC, Japan, paste)—performs better, as well as to determine the most effective preparation method among sandpaper (control), 36% orthophosphoric acid (H_3_PO_4_), sandblasting, and 9.5% hydrofluoric acid (HF). The tested gum-imitating material was Amaris Gingiva (VOCO, Germany). The connection between the composites was evaluated using a Z005 (Zwick-Roell) universal device. Surface tests were carried out using an SJ-410 (Mitutoyo) profilometer. Evaluation of the prepared surface structures was performed using scanning electron microscopy (HITACHI S-4700). Etching with HF significantly improved the shear bond strength between composites. Sandblasting also enhanced the adhesion results, but the H_3_PO_4_ group achieved comparable results to the control group. However, since HF is not recommended for intraoral use, sandblasting (30 μm aluminum oxide particles applied with three passes at constant speed under a pressure of 2 bar from 1.5 cm) appears to be the most suitable clinical alternative.

## 1. Introduction

Dental caries is the most widespread noncommunicable disease globally [1]. Especially, in patients with gingival recession, the risk of tooth decay is high due to exposed root surfaces covered only by cementum without a protective enamel layer [2]. Other lesions such as erosion, abrasion, abfraction can cause class V cavities and are more likely to occur nowadays because of the increasing amount of patients with bruxism and high consumption of acidic drinks [3,4,5,6]. The etiology of these conditions appears to be multifactorial and a holistic approach is needed [3,7,8]. Additionally, aesthetics is of increasing importance for these patients, which can be obtained by using a gingiva-colored composite.

Gingival recession is a prevalent clinical condition associated with the increasing frequency of orthodontic treatments and bruxism [9,10]. In such cases, achieving an aesthetically acceptable dental filling is a real challenge because of the exposure of dental cementum. Both conservative and surgical approaches can be employed. Traditional dental composite is tooth-colored, which causes artificial elongation of the crown. Surgical treatment of the recession is very expensive, demanding high cooperation from the patient, and is a time-consuming procedure [11,12].

To obtain a less invasive and aesthetic method, gum-imitating composites can be used. These materials can be adhesively bonded to pre-existing dental fillings to improve aesthetic appearance. Aesthetic improvement may motivate patients to replace old restorations and prevent secondary caries, which occur in up to 50–60% of clinical cases [13].

Currently, resin-based composites (RBCs) are the most frequently used material for dental restorations, replacing dental amalgam as the material of choice [14,15]. RBCs are composed of a resin matrix, typically made of methacrylate monomers (e.g., bis-GMA, TEGDMA, UDMA) which are chemically bonded to inorganic filler particles using a silane coupling agent, and photo-initiators such as camphorquinone are added [16,17].

In anterior teeth, the predominant type of cavity is class V, characterized by a high configuration factor (C-factor: 5) referring to high polymerization stress, which indicates the high importance of strong adhesion between two composite materials (e.g., tooth-colored composite and gum-colored composite) [18]. In cases of lowering the gumline, adhesion becomes even more difficult due to the presence of cementum instead of enamel around the dental filling. Considering the above factors, the adhesion between the conventional and gum-imitating composites may prove to be significant.

Currently, there is a lack of studies evaluating the bond strength between gum-colored composite and conventional composite. Although the methods used to improve surface adhesion when repairing old restorations include abrasive paper, air abrasion with Al_2_O_3_ particles, argon plasma, atmosphere plasma, diamond bur, air abrasion with silica-coated Al_2_O_3_ particles, phosphoric acid etching, laser, total-etch adhesive, self-etch adhesive, silane, and unfilled resin [13,14].

In this preliminary study, we aimed to compare the bond strength of gum-colored composite to two base materials: flowable resin-based composites or paste resin-based composites. We determined the most effective (resulting in the highest SBS) surface preparation method among sandpaper (grit size 600, control), 36% orthophosphoric acid (H_3_PO_4_), sandblasting, and 9.5% hydrofluoric acid (HF). The applied surface preparation methods were tested across the two above-mentioned composites before the placement of gum-imitating material to resolve whether they have a consistent effect in both base systems. To evaluate the bond strength of the specimens, a shear bond strength (SBS) test was performed. Scanning electron microscopy (SEM) was used to examine the surface morphology, and surface roughness parameters, i.e., Ra, Rq, Rz, and Vo, were measured using a surface contact profilometer.

The first null hypothesis was that the base composite type has no significant effect on the results. The second null hypothesis was that there is no significant difference in the shear bond strength results between the surface preparation methods.

## 2. Materials and Methods

**Figure 1:**
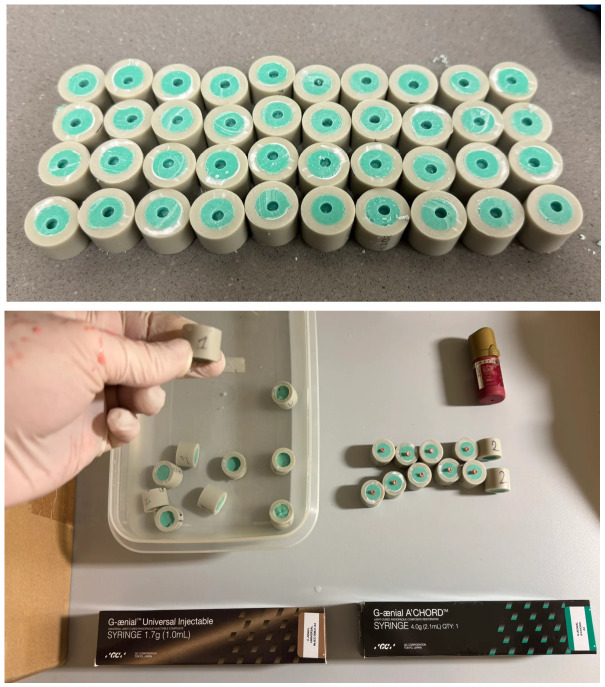
Acrylic forms in PVC tubes in the upper photo; in the lower photo: G-aenial Universal Injectable on the left and G-aenial A’CHORD on the right.

Acrylic resin was poured into 20 mm long, 21 mm diameter, and 4 mm wall thickness polyvinyl chloride (PVC) tubes and allowed to harden. A 6 mm deep cylindrical (Ø 5 mm) recess was then drilled into each acrylic sample. A total of 22 acrylic forms were randomly divided into two groups (n = 11). In the first group, the cavities were filled with a flowable resin composite (G-aenial Universal Injectable, GC, Tokyo, Japan). In the second group, paste resin composite (G-aenial A’CHORD, GC, Tokyo, Japan) was used. Then, all samples were finished and standardized using 600 grit sandpaper (Figure 1).

**Figure 2:**
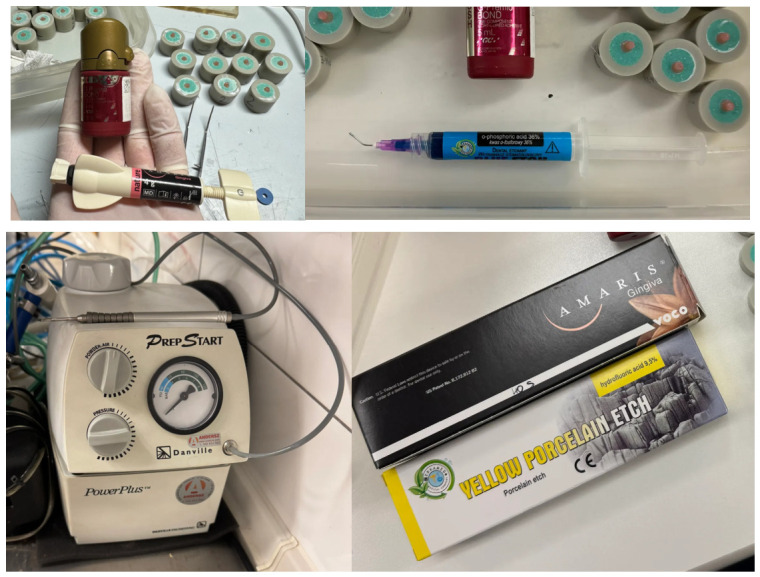
Control group in the top-left corner; H_3_PO_4_ agent in the top-right corner; sandblaster in the bottom-left corner; gum-colored composite and HF in the bottom-right corner.

The specimen surfaces were prepared separately, with 22 samples in each group. The first group was the control—no additional surface treatment was applied except standardization with sandpaper size 600. In the second group, the surfaces were etched with 36% orthophosphoric acid (H_3_PO_4_) for 30 s. The H_3_PO_4_ was rinsed off with water and then the water was blown away using an air-pressure blow tube. The third group underwent sandblasting using an air abrasion device (Power Plus Air Booster, Danville, CA, USA) with 30 μm aluminum oxide particles, applied with three passes at constant speed under a pressure of 2 bar and from a distance of 1.5 cm [19]. Residual dust was removed using an air-pressure blow tube. In the fourth group, the surfaces were treated with 9.5% hydrofluoric acid for 60 s. The HF was rinsed off with water and then the water was blown away using an air-pressure blow tube (Figure 2).

**Figure 3:**
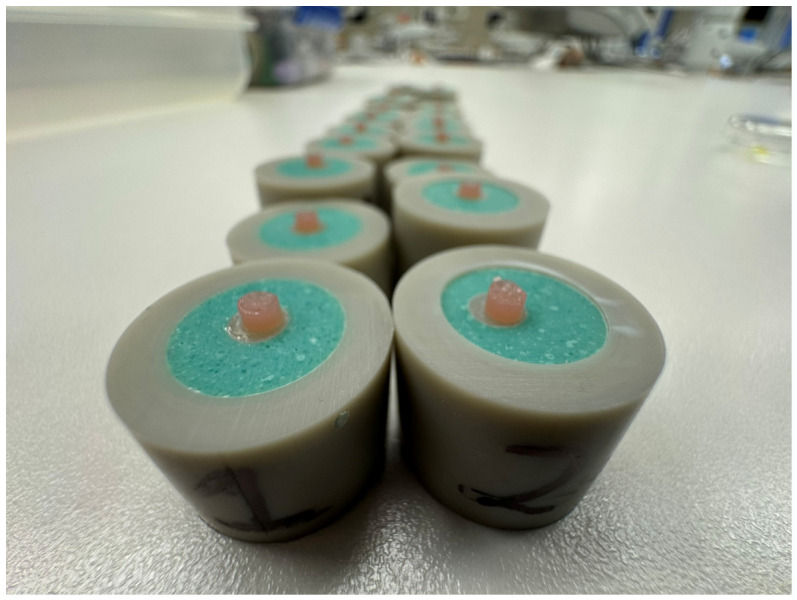
Amaris Gingiva—gum-imitating composite placed on the base specimens.

All specimen surfaces were treated with a bonding system (G-Premio BOND, GC, Aichi, Japan, pH = 1.5), which has performed effectively in other studies [20]. The bonding agent was applied after rising with water on dried surfaces and light-cured for 20 s with average power 1240 mW/cm^2^ (The Cure, Spring Health Products Inc., Norristown, PA, USA) according to the manufacture’s instruction. Subsequently, the tested gum-imitating composite material (Amaris Gingiva, VOCO, Cuxhaven, Germany) was placed over the adhesively prepared area and light-cured for 40 s. To ensure repeatable applications, silicone molds with a 3 mm diameter hole (mold thickness 1.5–2.0 mm) were used (Figure 3).

Additionally, in the early stage of the study, a universal bonding agent (OptiBond Solo Plus, Kerr, Scafati, Italy), which does not contain a strong acid in its composition, was tested and compared with a low-pH bonding agent. However, it was rejected from further analysis because it exhibited an unacceptably high standard of deviation in the control test.

All samples were immersed in distilled water for 24 h and measured for shear bond strength. The SBS tests were carried out using a Zwick Z005 (Zwick-Roell, Ulm, Germany) universal testing machine.

The surface morphology of the samples was examined using scanning electron microscopy (SEM, Hitachi S-4700, Tokyo, Japan) coupled with Energy-Dispersive X-ray Spectroscopy (EDS, Thermo Scientific UltraDry, Waltham, MA, USA). The SEM-EDS analysis was carried out using the following operating parameters: accelerating voltage—25 kV; and magnification—500–3000× (high-resolution mode). Prior to analysis, the samples were coated with a thin layer of carbon using a Cressington 108 Carbon/A sputter coater (Fujian, China). Observations were based on a few areas of a single sample in each group, and there were eight samples in total. The samples were prepared separately using specific surface preparation methods.

**Figure 4:**
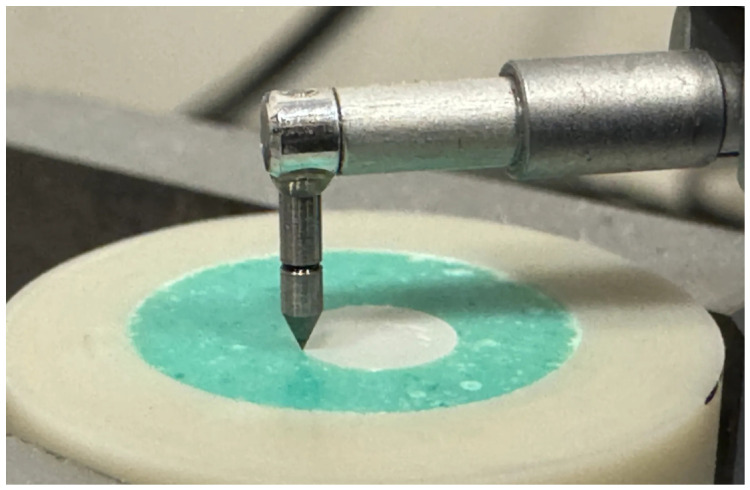
Surface test—the needle.

Surface roughness was measured using a contact profilometer SJ-410 (Mitutoyo, Kawasaki, Japan) with an accuracy of 0.1 μm. A 4 μm radius stylus moved linearly across the examined composite specimens. Each group of specimens was analyzed in three different directions, and the average value was calculated (Figure 4).

Statistical analysis was performed to evaluate the differences between the studied groups using the Statistica v. 13 program (StatSoft, Kraków, Poland). The normality of data distribution was assessed using the Shapiro–Wilk test. For variables demonstrating a normal distribution, multiple group comparisons were conducted using one-way analysis of variance (ANOVA). When the assumption of normality was not met, Kruskal-Walls test was applied. For pairwise comparisons between groups, Student’s *t*-test was used for normally distributed variables, while the Mann–Whitney U test was applied for variables that did not follow a normal distribution. The level of statistical significance was set at *p* = 0.05. For the analysis of qualitative parameters (the type of failure), the chi-square test was used.

## 3. Results

### 3.1. Shear Bond Strength (SBS)

For the shear bond strength, the highest values were recorded in the HF group—19.27 ± 3.29 MPa. Sandblasting improved the shear bond strength—17.49 ± 3.29 MPa. The H_3_PO_4_ group obtained results (15.47 ± 3.18 MPa) similar to those of the control group (16.32 ± 3.37 MPa). For flowable resin composite (G-aenial Universal Injectable), the following results were obtained: the control group exhibited an average shear bond strength of 16.24 MPa (±3.68); samples treated with H_3_PO_4_ showed 16.80 MPa (±3.62); sandblasting resulted in 18.56 MPa (±2.80); and treatment with HF achieved 19.32 MPa (±2.97). For paste resin composite (G-aenial A’CHORD): the control group demonstrated an average shear bond strength of 16.41 MPa (±3.06); treatment with H_3_PO_4_ resulted in 14.15 MPa (±2.74); sandblasting yielded 16.41 MPa (±3.79); and HF application led to 19.23 MPa (±3.62) (Table 1). The overall average values recorded for the flowable composite was 17.73 MPa (±3.42) and the paste material was 16.55 MPa (±3.69).

There was a statistically significant difference only for the paste between the HF and H_3_PO_4_ groups (*p* = 0.021115).

**Figure 5:**
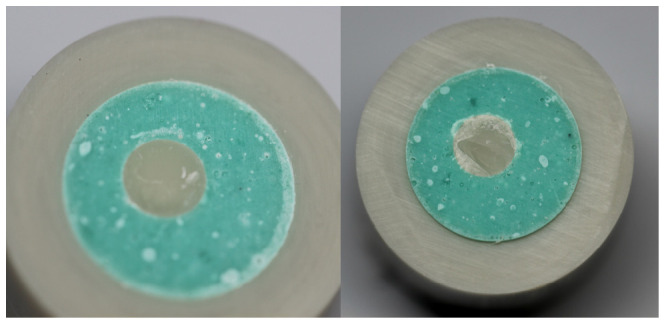
Representative photos of adhesive failure on the **left**; and cohesive failure on the **right**.

Additionally, the type of failure (adhesion, cohesion, mixed) was evaluated. In the paste specimens, failures were predominantly adhesive. In the flow specimens, the results were diverse, as in the H_3_PO_4_ group, various types of failures were observed; however, in 7 out of 11 specimens, the failure additionally occurred within the gum-colored composite. After sandblasting, adhesive failures with cohesive features were observed. In the HF group, 6 out of 11 samples showed purely adhesive failures, 2 out of 11 showed mixed adhesive failures with a failure breakage in the GC composite, and for 2 out of 11 the failures combined adhesive and cohesive failure in the GC composite with cohesive failure in the gum-colored composite (Figure 5). A chi-square test of independence showed a significant association between the type of failure and the tested groups, x^2^(14) = 44.51, *p* > 0.001 (Table 2).

**Table 1:** The mean, standard deviation, median, minimum, maximum, lower, and upper quartile of SBS [MPa] between the two types of composites with gum-imitating composite.

Surface Preparation	Mean	Standard Deviation	Median	Minimum	Maximum	Lower Quartile	Upper Quartile
Flowable composite							
Control	16.24	3.68	15.00	11.20	23.50	14.60	20.00
H_3_PO_4_	16.80	3.62	17.10	12.00	21.60	12.60	20.50
Sandblasting	18.56	2.80	17.90	15.40	25.10	15.90	20.30
HF	19.32	2.97	18.90	15.00	26.40	16.80	20.90
Optibond	19.19	6.02	17.90	9.52	28.10	13.80	24.80
Paste composite							
Control	16.41	3.06	17.10	8.26	19.30	15.50	18.50
H_3_PO_4_	14.15 *	2.74	13.80	9.20	19.20	12.50	15.30
Sandblasting	16.41	3.79	16.60	10.00	23.20	14.80	19.10
HF	19.23 *	3.61	18.40	13.60	26.70	16.90	22.60
Optibond	15.59	4.70	14.20	9.48	24.40	10.70	19.00

* statistically significant difference.

**Table 2:** Types of failure.

	Adhesive	Cohesive	Mixed
Flowable composite			
Control	0	1	10
H_3_PO_4_	2	0	9
Sandblasting	0	0	11
HF	6	0	5
Paste composite			
Control	6	0	5
H_3_PO_4_	11	0	0
Sandblasting	7	0	4
HF	7	0	4

### 3.2. Scanning Electron Microscope (SEM)

**Figure 6:**
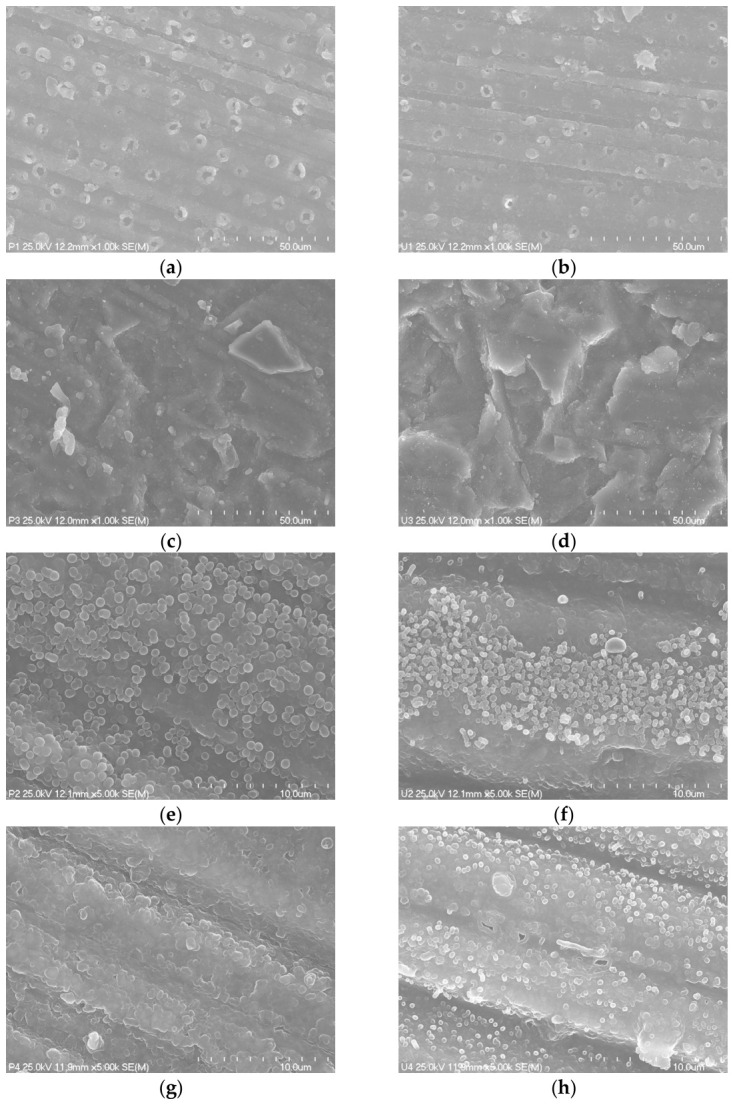
Scanning electron microscopy (SEM) micrographs of composites with controls and surface preparations ((**a**,**c**,**e**,**g**)—paste composite, (**b**,**d**,**f**,**h**)—flowable composite): ((**a**,**b**)—etched with 9.5% HF for 60 s), ((**c**,**d**)—sandblasted, 30 μm Al_2_O_3_, 6 bar), ((**e**,**f**)—etched with 36% H_3_PO_4_ for 30 s), and ((**g**,**h**)—control, sandpaper 600 grid), (**a**–**d**) at 1000×, (**e**–**f**) at 5000×). Figures (**a**,**b**) have increased brightness and (**e**–**h**) increased contrast.

Microscope analysis of the examined resin composite surfaces (Figure 6), etched with 9.5% HF for 60 s, at 1000× magnification revealed a structure distinct from the control surfaces.

Multiple circular regular pores were observed, which drained deeply (Figure 6a,b). The pores were approximately 3 µm in diameter. Some of them were surrounded by grains of dissolved material. Between these pores, areas of smooth surface similar to the control specimen were noticed. In the paste resin composite specimen, the pores were noticeably bigger, more visible, and more numerous than in the flowable one (Figure 6a,b).

Specimens sandblasted with 30 μm Al_2_O_3_ exhibited the greatest surface roughness compared to the other groups (Table 3). In the SEM images of the specimens, sharp-edged structures of irregular shapes and sizes were predominant (Figure 6c,d). Elevated areas of varying height and separated by valleys of varying depths were observed. Partially exposed filler particles were also noticeable.

Examination of specimens etched with 36% H_3_PO_4_ for 30 s revealed a structure similar to the sanded specimens with sandpaper grit 600. At 5000× magnification, areas of increased roughness were observed—the surface of the etched specimens appeared less conglomerated, with partially exposed spherical filler particles, compared with the control specimens (Figure 6e,f).

Control group specimens presented a smooth surface with conglomerated material along longitudinal grooves. The structure was more regular compared to the other groups (Figure 6g,h).

### 3.3. Surface Roughness Test

Three measurements per sample were performed with a contact profilometer. Nine parameters were calculated by an average of these three measurements: arithmetic average roughness (Ra), root mean square roughness (Rq), maximum height of the profile (Rz), mean width of the profile elements (RSm), profile length ratio (Rlr), profile length of the ordinate (Rlo), maximum profile peak height (Rp), maximum profile valley depth (Rv), and oil volume parameter (Vo) (Table 3). The highest Ra, Rq, Rz, Rsm, Rlr, Rlo, Rv, and Vo were noticed in the sandblasted surface. The Ra parameter for sandblasting was around 5.8 times higher than for etching with HF in both groups (flowable and paste composite). Statistically significant difference occurred only in: Ra—paste (H_3_PO_4_ and sandblasting, *p* = 0.033215); Rq—paste (H_3_PO_4_ and sandblasting, *p* = 0.033215); Rz—paste (H_3_PO_4_ and sandblasting, *p* = 0.033215); Rlr—flowable (control and sandblasting, *p* = 0.039470); Rlr—paste (H_3_PO_4_ and sandblasting, *p* = 0.023313); Rlo—flowable (control and sandblasting, *p* = 0.039470); Rp—paste (H_3_PO_4_ and sandblasting, *p* = 0.033215); Rv—paste (H_3_PO_4_ and sandblasting, *p* = 0.01617); and Vo—flowable (sandblasting and HF, *p* = 0.013407) and paste (H_3_PO_4_ and sandblasting, *p* = 0.019445).

**Table 3:** Roughness parameters depending on the material and the method of preparing its surface.

	Ra [µm]	Rq [µm]	Rz [µm]	RSm [µm]	Rlr	Rlo [mm]	Rp [µm]	Rv [µm]	Vo
Flowable composite									
Control	0.1773 ±0.077	0.2460 ±0.121	1.5703 ±0.687	117.1 ±26.83	1.0017 ±0.001 *	0.8013 ±0.001 *	0.8913 ±0.653	0.6790 ±0.097	0.0021 ±0.0004
H_3_PO_4_	0.1410 ±0.033	0.1810 ±0.045	1.2357 ±0.519	95.1667 ±95.58	1.0027 ±0.003	0.8023 ±0.002	0.4493 ±0.120	0.7863 ±0.407	0.0018 ±0.0002
Sandblasting	1.4130 ±0.40	1.9403 ±0.478	9.8947 ±1.843	126.4 ±18.27	1.0290 ±0.005 *	0.8233 ±0.004 *	2.4240 ±0.643	7.4703 ±1.482	0.0666 ±0.0197 *
HF	0.2420 ±0.007	0.4460 ±0.002	3.4433 ±0.309	X	1.0040 ±0	0.8030 ±0	2.7360 ±0.262	0.7073 ±0.047	0.0014 ±0.0002 *
Paste composite									
Control	0.1457 ±0.008	0.2297 ±0.029	1.9820 ±0.256	114.4 ±0	1.0027 ±0.001	0.8020 ±0	1.1910 ±0.293	0.7907 ±0.037	0.0020 ±0.0004
H_3_PO_4_	0.1310 ±0.022 *	0.1910 ±0.063 *	1.4783 ±0.695 *	53.8 ±9.48	1.0020 ±0	0.8017 ±0.001	0.7677 ±0.661 *	0.7107 ±0.033 *	0.0015 ±0.0003 *
Sandblasting	1.4820 ±0.419 *	2.2083 ±0.718 *	12.1473 ±3.666 *	196.567 ±72.36	1.0277 ±0.005	0.8220 ±0.005	2.9213 ±1.031 *	9.2257 ±2.728 *	0.0634 ±0.0210 *
HF	0.2563 ±0.011	0.3880 ±0.004	3.0013 ±0.187	171.5 ±0	1.0040 ±0	0.8037 ±0.001	1.6450 ±0.158	1.3563 ±0.344	0.0024 ±0.0005

Ra—arithmetic average roughness, Rq—root mean square roughness, Rz—maximum height of the profile, RSm—mean width of the profile elements, Rlr—profile length ratio, Rlo—profile length of the ordinate, Rp—maximum profile peak height, Rv—maximum profile valley depth, Vo—oil volume parameter; * statistically significant difference. X—ERROR in measurements.

## 4. Discussion

Primarily, gingiva-colored composites were used to improve the aesthetics of titanium implants and zirconium or porcelain crowns [21,22,23,24,25]. For titanium and gingiva-colored composite connections, SBS results show 4.35 ± 1.54 MPa [26] and up to 20.8 ± 2.4 MPa with metal link primer use, and 15.1 ± 1.4 MPa for zirconium [24]. The thermal aged group obtained only half of the SBS for titanium, zirconia, and modified PEEK compared to the non-aged group, obtaining up to 8.89 ± 3.01 MPa, which could not be sufficient in the same clinical cases [27]. Porcelain and zirconia can be characterized with gingival shades in the laboratory, which can be considered a more consistent method.

Gingiva-colored composites can be used not only for caries cases but also in non-carious cervical lesion cases. In coffee, wine, or sour drink consumers with erosions, gingiva-colored composites can be used primarily to treat the same cavities as traditional composites. Referring to the aesthetic aspect, gingiva-colored composites can be more susceptible to staining in coffee and wine compared to tooth-colored composite [28]. Abfraction cavities are usually located in the cervical region, near the gumline, where occlusal forces are concentrated. Therefore, the use of a gingiva-colored composite may be justified in some cases [29,30].

According to increasing aesthetic demands placed on dental fillings, there is a need to examine the adhesion strength of gum-imitating composites and traditional composites to improve the aesthetic properties of dental restorations. High adhesion strength to existing fillings is even more important in root cavities because cementum does not have as good adhesion as enamel (36.19 ± 1.4 MPa in SBS test) [31]. As a result, most of the functional forces are concentrated at the interface between the traditional composite and the gum-colored composite. Studies have suggested that the bond strength between two traditional composites around 15–30 MPa can be considered as clinically satisfactory, referring to clinically acceptable enamel–composite connections [32,33]. In cases of insufficient clinical adhesion strength, extending the cavity to the dentine level can be recommended, for which an SBS value of 49.38 ± 1.23 MPa has been reported [31].

During placement of composite restorations, the bonding between successive layers of applied and polymerized material depends on the incomplete conversion of the polymer matrix and on the presence of an oxygen-inhibition layer, which is subsequently removed during finishing and polishing of the existing restoration. Mechanical finishing also eliminates the silane layer from the filler surface, thereby preventing the formation of a chemical bond between the filler, which is a major part of the restoration, and the polymer matrix of the gum-imitating composite used for the repair or improvement of aesthetics. Under these circumstances, the composite–composite bond is mainly governed by the remaining free methacrylate groups in the polymer matrix of the existing restoration, which can react with dimethacrylates in the matrix of the repair composite. As the composite ages, the polymer undergoes degradation, including a reduction in the concentration of reactive methacrylate groups, which limits the potential to obtain a sufficient bond strength. It has been reported that the repair strength was 48% of the cohesive strength with a bonding agent used. Most of the bond strength decreased within first 24 h, so it is better to attach gum-colored composite soon after traditional composite placement [34].

Because the bond between the polymer matrix of the existing restoration and the new composite may be insufficient, it is recommended to increase the surface roughness of the restoration by creating mechanical micro retentions [21]. Equally important is the use of materials with adequately low viscosity, capable of thoroughly wetting the surface and, where possible, forming chemical bonds not only with the resin matrix but also with the exposed filler surface [35].

Regarding roughness parameters, Ra is the most commonly used parameter for assessing surface quality in restorative dentistry. As an average roughness parameter, it provides a general clinical overview, but it has to be underlined that different topographies can produce the same Ra results. This number averages all the absolute values of the profile deviation from the mean line. A situation may arise where dense and sharp peaks will have the same Ra as a gently undulating surface if their average distance from the mean line is the same. It is therefore necessary to supplement this parameter with the additional included parameters to better understand the character of the surface morphology, such as RSm, Rq, Rz, and Vo. RSm represents the mean width between peaks and valleys which may influence how effectively a bonding system can flow across the surface. Vo may also be useful for assessing volume, indicating how much liquid can be retained within the gaps [36,37]. For comparison, the mean Ra value reported in the study using 5 s sandblasting (with different angulation, powder type, pressure) was 0.13 on average [38]. One study reported an average Ra value of 0.185 [39].

The surface preparation methods demonstrated significant differences in SBS outcomes. The best result in the SBS test was noticed in the HF surface preparation method, but HF is not recommended for intraoral use due to its toxicity [40]. However, it can be applied in the laboratory during the manufacturing process. SEM analysis showed that HF creates deep canal-like pores. This qualitative observation corresponded with the surface test results, where HF produced higher roughness values (Ra, Rz, RSm) compared with the control and H_3_PO_4_ groups. The bonding agent may penetrate these micro-canals thanks to its low viscosity, which is consistent with the high SBS values. The higher RSm value may facilitate better penetration of the bonding agent into these canals. However, SEM observations demonstrated that sandblasting produced the highest developed surface among all evaluated materials. This is also confirmed by the surface test results, which showed multiple times higher values of surface roughness profiles for the sandblasted specimens, while the corresponding parameters for the other groups (control, etched with HF and H_3_PO_4_) remained at comparable levels. The inconsistency between the high SBS test results and the relatively moderate surface test values in the HF group can be explained by the fact that a 4 μm diameter needle was used, which may have been too wide to enter the small HF-induced canals, while the low-viscosity bonding resin can still infiltrate them effectively. In Sugai et al.’s research, HF treatment yielded the highest results in terms of SBS and surface free energy [41].

The method using sandblasting reached the second highest result in the SBS test and it can be used intraorally. Sandblasting expands the surface mechanically through breaking off parts of the material in various sizes. It effectively increases the available surface for adhesion. Additionally, it creates many sharp edges in various angles, which can provide micromechanical interlocking for the material flowing into the created spaces. It can be easily used in a dental office. Another improvement to this method could be the use of the CoJet system, which uses abrasion with silanized aluminum oxide particles [42]. However, it has been proven that the sandblasting of dentine may reduce SBS [43,44], so it is recommended to use sandblasting only for composites in areas where gingiva-colored composite will be attached.

H_3_PO_4_-exposed filler compounds visible in SEM may improve adhesion. However, in the SBS test, the observed results were significantly worse in comparison to the HF and sandblasting groups. The surface test confirmed worse roughening of the surface by H_3_PO_4_. While etching dentine with 37% H_3_PO_4_ for 30 s is more justifiable in the context of placing a new restoration, it is not a particularly effective method for improving adhesion to existing restorations [45].

In comparison, the highest achieved SBS result for gingiva-colored composite with titanium, zirconia, and modified PEEK was 16.86 ± 3.56 MPa [27].

This study fills an important gap in the literature by investigating the adhesion between gum-colored composite and conventional composite, and by comparing flowable and paste materials in this context. The absence of directly comparable studies on gingiva-colored composite adhesion limits the possibility of benchmarking the obtained values with previously published results. The specimens were stored in distilled water for 24 h, but there was no intraoral simulation of temperature fluctuations, enzymatic activity, pH changes, or long-term aging (thermocycling). Therefore, the results of this study should be regarded as in vitro indicators under controlled conditions rather than direct predictors of in vivo behavior.

From a future perspective, combining two surface preparation methods, such as sequential HF and H_3_PO_4_ etching applied to the same surface, may further enhance adhesion strength by integrating their complementary micromechanical and chemical effects [46]. It is noted that laser application effectively roughened the composite surface for repair, while the diamond bur produced the highest shear strength. Both methods can be considered for use in future studies [21,47]. Different settings of sandblasting may be considered for examination. However, according to existing studies on traditional composites, no significant differences in SBS have been demonstrated when varying sandblasting parameters such as air pressure, distance, tip size, particle size, and angulation applied during restoration repair [44].

## 5. Conclusions

In this study, sandblasting proved to be the most effective, clinically applicable, method for roughening the composite surface in order to adhesively attach gum-colored composite to an existing traditional composite restoration. Although the HF group achieved the highest bond strength values, HF is not recommended for intraoral use, whereas the H_3_PO_4_ group showed results comparable to those of the control group.

## Data Availability

The original contributions presented in this study are included in the article. Further inquiries can be directed to the corresponding author.

## Abbreviations

The following abbreviations are used in this manuscript:

RBC	Resin-based composite
Bis-GMA	Bisphenol A glycidyl methacrylate
TEGDMA	Triethylene glycol dimethacrylate
UDMA	Urethane dimethacrylate
Al_2_O_3_	Aluminum oxide
H_3_PO_4_	Orthophosphoric acid
HF	Hydrofluoric acid
SBS	Shear bond strength
SEM	Scanning electron microscope
ANOVA	Analysis of variance
Rq	Arithmetic roughness
Rz	Maximum height of the profile
RSm	Mean width of the profile elements
Rlr	Profile length ratio
Rlo	Profile length of the ordinate
Rp	Maximum profile peak height
Rv	Maximum profile valley depth
Vo	Volume parameter
